# SNP discovery in apple cultivars using next generation sequencing

**DOI:** 10.1186/1753-6561-5-S7-P42

**Published:** 2011-09-13

**Authors:** Sérgio Alencar, Orzenil Silva-Junior, Roberto Togawa, Marcos Costa, Luís Fernando Revers, Georgios Pappas

**Affiliations:** 1 Embrapa Recursos Genéticos e Biotecnologia - Parque Estação Biológica - Av. W5 Norte (final) Caixa Postal 02372 - Brasília, DF - 70770-917, Brazil; 2 EMBRAPA Uva e Vinho - Rua Livramento, 515 - Caixa Postal 130 - Bento Gonçalves, RS - 95700-000, Brazil

## Background

Knowledge about single nucleotide polymorphism (SNP) markers is extremely important in the development of genotyping assays, allowing improvements in plant breeding through marker-assisted selection. With the emergence of next generation sequencing platforms, high-density SNP discovery in the genome of plant crops becomes more achievable. In this project, we carried out whole genome resequencing of two apple cultivars (M13/91 and Fred Hough, developed by Epagri [1,2]) using the recently released draft genome sequence of the domesticated apple genome (*Malus* x *domestica* Borkh.) [3] as a reference for SNP discovery.

## Materials and methods

We sequenced four DNA samples (two from M13/91 and two from FH cultivars) on four lanes of the Illumina GA II platform (single-end sequencing), with the Illumina PhiX sample used as a control. Image analysis and base calling were performed using on-instrument real time analysis, after which the Off-Line Basecaller was used to convert per-cycle base call files (.bcl) into per read base call files (.qseq). The resulting short reads were aligned to the reference genome with the BWA software [4], with the maximum edit distance set to 3, quality threshold for read trimming set to 15, and no gap opens allowed. Next, SAMtools [5] utilities were used to convert SAM into BAM format, to remove read duplicates and indels, and also to sort reads by coordinates. SNP discovery was then carried out using the GATK Unified Genotyper SNP caller [6].

## Results

Alignment of the M13/91 and FH reads to the reference genome resulted in a total of 37,757,897 and 60,400,252 reads mapped, respectively. Considering only reads with root-mean-square mapping quality greater than 20, and occurring at raw read depths (DP) greater than 6 and lower than 20, a total of 143,468 and 474,483 heterozygous putative SNPs were identified when comparing the reference genome with the M13/91 and FH cultivars, respectively. A total of 80,554 heterozygous putative SNPs are shared by both M13/91 and FH cultivars. When considering only homozygous putative SNPs, a total of 20,296 (M13/91) and 70,659 (FH) SNPs were identified. A search was also made between the M13/91 and FH cultivar genomes, resulting in a total of 2,631 SNPs which are homozygous in FH and heterozygous in M13/91, and 4,768 SNPs which are homozygous in M13/91 and heterozygous in FH. In order to determine whether the differences in SNP frequencies between these cultivars are due to differences in read coverage obtained from sequencing, we set up a cut-off value above which all SNP calls in both cultivars had the same coverage, and it showed that their SNP frequency is similar.

## Conclusions

We have used next generation sequencing data combined with high-density SNP detection methods to discover large numbers of putative SNPs in apple cultivars, which can be used in the development of genotyping assays.
